# Construction and validation of a prognostic model for osteosarcoma patients based on autophagy-related genes

**DOI:** 10.1007/s12672-022-00608-9

**Published:** 2022-12-31

**Authors:** Biao Ning, Yixin Liu, Tianzi Xu, Yi Li, Dongyi Wei, Tianhe Huang, Yongchang Wei

**Affiliations:** Department of Radiation and Medical Oncology, Zhongnan Hospital of Wuhan University, No. 169 Donghu Road, Wuchang District, Wuhan, 430072 Hubei Province People’s Republic of China

**Keywords:** Osteosarcoma, Autophagy-related gene, Metastasis, Prognosis

## Abstract

**Background:**

Osteosarcoma is the most frequent primary bone malignancy with a poor prognosis because of pulmonary metastasis. Autophagy is strongly associated with tumor metastasis, and it is valuable to construct an autophagy-related gene risk model for predicting the prognosis of osteosarcoma.

**Methods:**

We obtained ARGs from the Human Autophagy Database and RNA-sequencing data of osteosarcoma patients from the Gene Expression Omnibus (GEO) database. Subsequently, univariate and multivariate cox regression analyses were performed to construct a three-gene prognostic model and its accuracy was further confirmed in the Therapeutic Applications Research to Generate Effective Treatments (TARGET) database. Afterward, we detected the expression levels and effects on osteosarcoma cells metastasis of MYC and MBTPS2, which were involved in the model.

**Results:**

In both training and verification cohorts, patients with lower risk scores had longer OS, and the model was identified as an independent prognostic factor in osteosarcoma. Besides, the ROC curve demonstrated the reliability of the model. Furthermore, RT-qPCR, Western Blotting and IHC results indicated that MYC and MBTPS2 were differently expressed in osteosarcoma tissues and cell lines. MYC knockdown or MBTPS2 overexpression prevented the capacity of migration and invasion in osteosarcoma cell lines through inhibiting cellular autophagy.

**Conclusion:**

The risk model based on three ARGs had a strong ability to predict the prognosis of osteosarcoma patients. Our findings also suggested that MYC and MBTPS2 were two major factors regulating autophagy in osteosarcoma, and could serve as potential therapeutic targets for osteosarcoma.

**Supplementary Information:**

The online version contains supplementary material available at 10.1007/s12672-022-00608-9.

## Introduction

Osteosarcoma, often occurring in children and adolescents, is the most common primary and highly aggressive bone tumor [1]. Worldwide, the incidence peaks of osteosarcoma in boys aged 15 to 19 years and in girls aged 10 to 14 years [2]. Osteosarcoma is mainly composed of malignant osteoblasts, which can produce immature bone and bone tissue [3]. Although more than two-thirds of patients with locally diseased osteosarcoma are likely to have longer survival, the prognosis is generally poor in patients with metastatic or recurrent osteosarcoma or those older than 40 years [4]. Treatment options such as neoadjuvant chemotherapy and limb-salvage surgery have advanced tremendously over the past few decades [5, 6]. However, the 5-year survival rate for locally diseased osteosarcoma patients remains at 60–70%, while for metastatic and recurrent patients is less than 20% [7, 8]. Therefore, it is imperative to find effective prognostic biomarkers for risk assessment of osteosarcoma patients to improve their prognosis and overall survival.

Autophagy is a traditional intracellular lysosome-dependent degradation pathway that normally engulfs and digests damaged organelles and excess proteins to provide nutrients or ATP for cell survival in response to extracellular and intracellular stress [9]. It has been reported that abnormal autophagy was associated with the occurrence and development of various diseases, including malignant tumors [10, 11]. In recent years, it was gradually recognized that autophagy played vital roles in various tumors, including breast cancer, non-small cell lung cancer, and glioma [12–14]. Likewise, autophagy also affects osteosarcoma progression. Some tumor suppressor genes frequently inactivated in osteosarcoma, such as RB1, PTEN or TP53 can regulate autophagy. Meanwhile, various oncogenes activated in osteosarcoma could also regulate autophagy. All of these findings suggest the dysregulation of autophagy in osteosarcoma [15]. Consequently, analysis and identification of ARGs are helpful to improve our understanding of the relationship between autophagy and osteosarcoma.

In this study, we explored the prognostic value of ARGs in osteosarcoma. Firstly, we obtained ARGs from the Human Autophagy Database (HADb) and RNA-sequencing (RNA-seq) data of osteosarcoma patients from the Gene Expression Omnibus (GEO) database. Subsequently, univariate and multivariate cox regression analyses were performed to construct a three-gene prognostic model, which had good prognostic value and could be used as an independent prognostic indicator in the GEO training cohort. In addition, this model was further confirmed in the Therapeutic Applications Research to Generate Effective Treatments (TARGET) database. Moreover, the ARGs in the model were verified in different cell lines by quantitative real-time PCR (RT-qPCR) and in osteosarcoma patients’ samples by immunohistochemistry (IHC). Finally, we found that MYC knockdown or MBTPS2 overexpression inhibited osteosarcoma migration/invasion through affecting autophagy.

## Materials and methods

### Data acquisition and processing

The mRNA sequencing file and corresponding clinical characteristics of osteosarcoma patients were downloaded from the GEO database (https://www.ncbi.nlm.nih.gov/geo/) and TARGET database (https://ocg.cancer.gov/programs/target). The list of ARGs was extracted from the human autophagy database (HADb, http://www.autophagy.lu/clustering/index.html). In this study, we defined osteosarcoma patients from the GSE21257 cohort (platform GPL10295, Illumina human-6 v2.0 expression bead chip Illumina, Inc., San Diego, CA, United States) as training cohort and the TARGET database as verification cohort. Patients who survived less than 90 days were excluded. The clinical features of the two cohorts are detailed in Table 1.

**Table 1:** Characteristics of patients in training and verification cohorts

Features	Training cohort (n = 53)	Verification cohort (n = 94)
Age		
≤ 16	25	55
> 16	28	39
Gender		
Male	34	55
Female	19	39
Metastasis		
Yes	34	22
No	19	72
Primary tumor site		
Arm/hand	8	7
Leg/foot	44	82
Other	1	5
Vital status		
Dead	23	37
Alive	30	57

### Construction of autophagy-related prognostic signature

To screen out ARGs associated with prognosis, we performed a univariate Cox proportional hazards analysis based on the criterion of P < 0.05. Then, an ARG-related risk model was generated by using a multivariate Cox hazard model analysis. To avoid overfitting, Akaike Information Criterion (AIC) was used to select the model step by step, and the model with the lowest AIC value (150.71) was considered the optimal model. The risk score formula was calculated as follows: Risk score = $\sum_{i=1}^{n}Coef(i)\times x(i)$, where Coef(i) represented the coefficient of a specific gene and x(i) indicated the expression level of the same gene.

### Risk model prediction ability in the training group and validation group

Patients in the training cohort were divided into high- and low-risk groups according to the median risk score. Kaplan–Meier survival analysis was used to assess the predictive ability of this model. Model reliability was assessed by time-dependent receiver operating characteristic (ROC) curve. To evaluate whether risk score was an independent predictor of prognosis in osteosarcoma patients, univariate and multivariate Cox regression analyses were carried out. In addition, correlations between clinical features and genes within the model were investigated. Kaplan–Meier survival curve and ROC curve were also drawn in the verification cohort to verify the stability and reliability of the risk model.

### Chemicals and reagents

Antibodies used for this study were as follows: GAPDH (Cat# 10494-1-AP) and E-Cadherin (Cat# 20874-1-AP) from Proteintech (Wuhan, China). MYC (Cat# 343250), MBTSP2 (Cat# 164301), Vimentin (Cat# R22775), N-Cadherin (Cat# 380671), MMP9 (Cat# 380831), LC3 (Cat# 381544), SQSTM1/p62 (Cat# 382862), and Beclin-1 (Cat# 381896) from ZENBIO (Chengdu, China). The endotoxin-free plasmid extraction reagent kit was purchased from TIANGEN (Cat# DP118, Beijing, China).

### Clinical sample and patients

Seven pairs of pathological tissues from patients diagnosed with osteosarcoma at Zhongnan Hospital of Wuhan University from September 2019 to December 2021 were collected for RT-qPCR and immunohistochemical (IHC) analysis. The study procedures were approved by Ethics and Scientific Committee (NO. LYL2021049) and all the patients provided written informed consent before tissue acquisition. The clinical information of these patients was showed in Table S1.

### Cell culture and transfection

Human osteoblast cell line (hFOB) and four human osteosarcoma cell lines (143B, MG63, HOS, U2OS) were from American Type Culture Collection (Manassas, USA). Cell culture medium and fetal bovine serum (FBS) were purchased from Hyclone (USA) and ABW (Shanghai, China), respectively. hFOB was cultured in DMEM/F12 medium with 20% FBS, 143B and HOS were cultured in MEM with 10% FBS, MG63 was cultured in DMEM medium with 10% FBS, and U2OS was cultured in Mycco’5A medium with 10% FBS. All cells were cultured with 100 μg/ml penicillin and streptomycin at 37 °C under an atmosphere of 5% CO2.

The small-interference RNA (siRNA) oligomers targeting MYC and MBTPS2 overexpressed plasmid were designed and synthesized by Tsingke Biotechnology (Beijing, China). 143B and HOS cells were seeded into 6-well culture plates at a density of 2 × 10^5^ cells/well and transfected with different siRNA oligomers (si-NC, si-MYC #1, si-MYC #2) using GP-Transfect-Mate reagent (GenePharma, Suzhou, China). The culture medium was removed 10 h after transfection and replaced with complete culture medium. After 24–48 h, 143B and HOS cells were collected for verification of MYC knockdown effects and subsequent experiments. Similarly, MG63 and U2OS cells (2 × 10^5^ cells/well) were plated in 6-well culture plates overnight and treated with the negative control (Vector) and MBTPS2 overexpression (OE) plasmids for 48 h according to the manufacturer's instructions. The siRNA oligomers sequences were showed in Table S2.

### RNA extraction and RT-qPCR

Total RNA of osteosarcoma patients’ specimens and different cell lines was extracted with Trizol reagent (Vazyme, Cat# R401-01, China), and cDNA was obtained by reverse transcription process using Hifair II Strand cDNA Synthesis Super Mix (Cat# 11120ES60, Yeasen, Shanghai, China) based on the product instructions. RT-qPCR was carried out with the CFX Connect Detector instrument (Bio-Rad, USA) and the relative mRNA expression level was calculated by the 2^−ΔΔCt^ method [16]. The primer sequences of genes were presented in Table S2.

### Protein isolation and Western blotting

Radioimmune precipitation assay (RIPA) buffer containing PMSF and protease inhibitor cocktail at a ratio of 100:1:1 was used to lyse cells for 30 min on ice, then the protein concentration was measured by a BCA Protein Assay Kit (Cat# P0012, Beyotime, Shanghai, China). In total, 20 μg protein were separated by SDS-PAGE gel, transferred to PVDF membranes (Cat# 05317, Millipore, USA), and blocked with protein-free rapid blocking buffer (Cat# PS108P, Epizyme, China) for 30 min at room temperature (RT). All the membranes were incubated with corresponding primary antibodies (dilution ratio, 1:1000) at 4 ℃ overnight. The next day, all blots were incubated with HRP-conjugated secondary antibodies for 1 h at RT. Immunoreactive proteins were visualized by chemiluminescence reagent (Cat # BLOG001, Biology, Wuhan, China) according to the manufacturer’s instructions.

### Immunohistochemistry analysis

Paraffin-embedded tissue samples were cut into consecutive 4 mm-thick sections. After dewaxing and rehydration, slides were incubated in 3% hydrogen peroxide for endogenous peroxidase activity blocking. Antigen repair was performed by microwave heating method and the slides were blocked with 5% goat serum albumin. Subsequently, they were incubated with primary antibodies overnight in a 4 °C wet box. The next day, after PBS washing 3 times, the slides were incubated with HRP-conjugated secondary antibody for 20 min at a 37 °C incubator. Immunoreactivity was detected using DAB chromogen and observed under the microscope (Olympus, Tokyo, Japan).

### Transwell assay

Boyden chamber transwell assay was used to detect the migration and invasion abilities of osteosarcoma cells. Briefly, after transfected with siRNA oligomers targeting MYC or MBTPS2 overexpressed plasmid for 48 h, 2 × 10^4^ osteosarcoma cells (143B, HOS, MG63, and U2OS) resuspended with serum-free medium were added into the upper chamber (Corning, Cat# 3422, USA) covered with or without Matrigel gel (BD, Cat# 356234, USA), while 500 μl medium containing 15% FBS was placed in the lower chambers. After incubation for 24 h, cell layer attached to the lower surface was fixed with 4% paraformaldehyde and stained with 0.1% crystal violet. An inverted phase-contrast microscope was used for observation and photography.

### Statistical analysis

All data were presented as the mean of three independent experiments, which were routinely performed in triplicate, and analysed using unpaired t-test or one-way ANOVA by using GraphPad Prism 8. 0 software. P < 0. 05 was regarded as statistically significant.

## Results

### Identification of ARGs with significant prognostic value in osteosarcoma

**Fig. 1:**
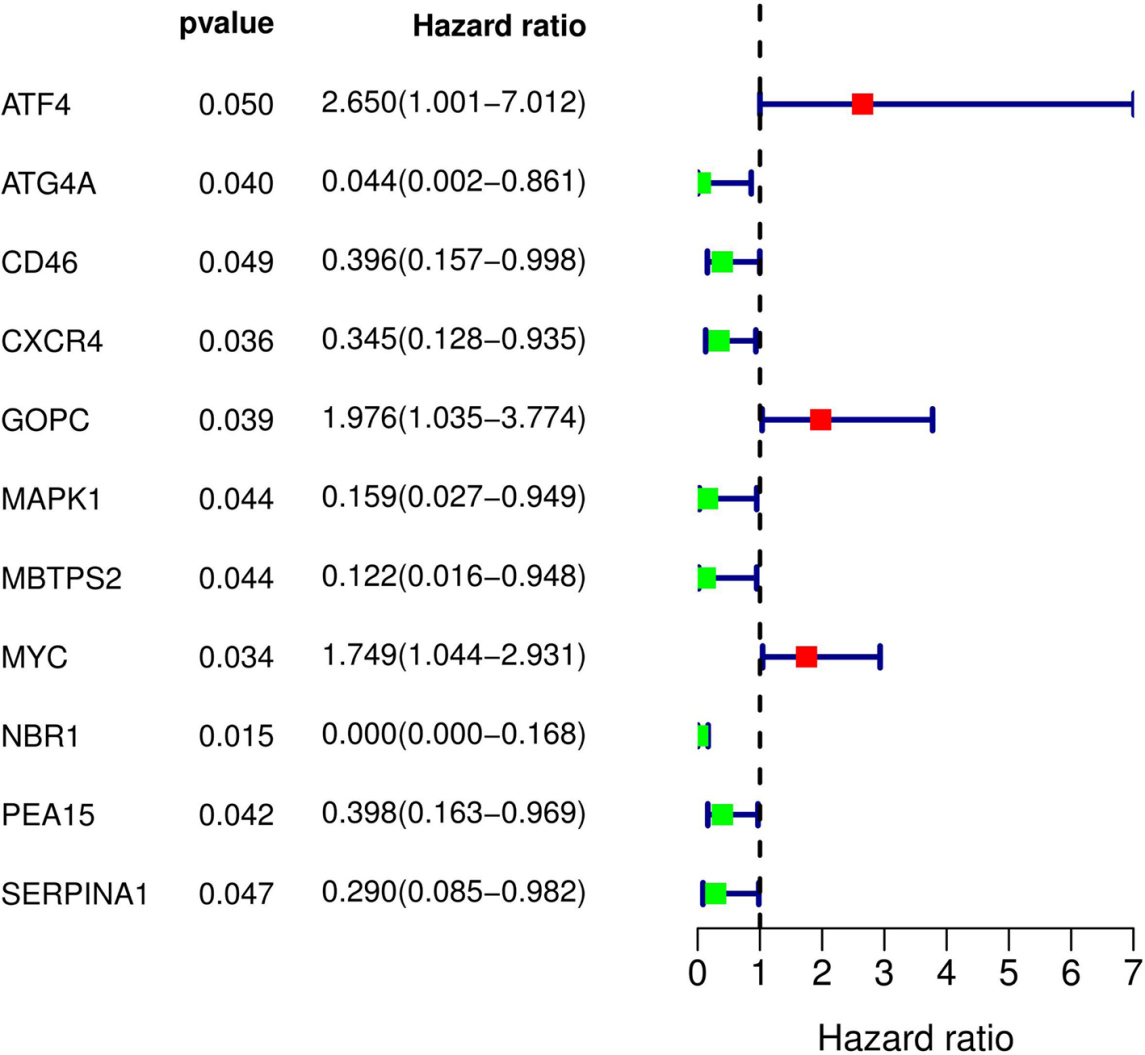
Identification of AGRs with significant prognostic value in osteosarcoma through univariate Cox proportional hazards analysis

A total of 11 ARGs were significantly associated with the survival of osteosarcoma patients in the training cohort (p < 0.05) by univariate Cox proportional-hazards analysis, including eight low-risk genes (hazard ration (HR) < 1) and three high-risk genes (hazard ration (HR) > 1) (Fig. 1). Subsequently, multivariate Cox analysis further screened out 3 genes, namely MYC, MBTPS2, and CXCR4, from the above 11 ARGs associated with prognosis. The optimal autophagy-related prognostic risk model was established through the three genes. The prognostic risk score formula of the model was as follows: prognostic score = (0.826029 × MYC) + (− 3.16546 × MBTPS2) + (− 1.07872 × CXCR4) (Table 2).

**Table 2:** The Multivariate Cox Regression Analysis of GSE21257

Gene	Coef	HR	HR.95L	HR.95H	p-value
CXCR4	− 1.07872	0.340029	0.129429	0.893306	0.028604
MBTPS2	− 3.16546	0.042195	0.003907	0.455679	0.009124
MYC	0.826029	2.284231	1.305081	3.997998	0.003825

HR, hazard ratio

### Validation of the prognostic performance of the ARGs signature in osteosarcoma

**Fig. 2:**
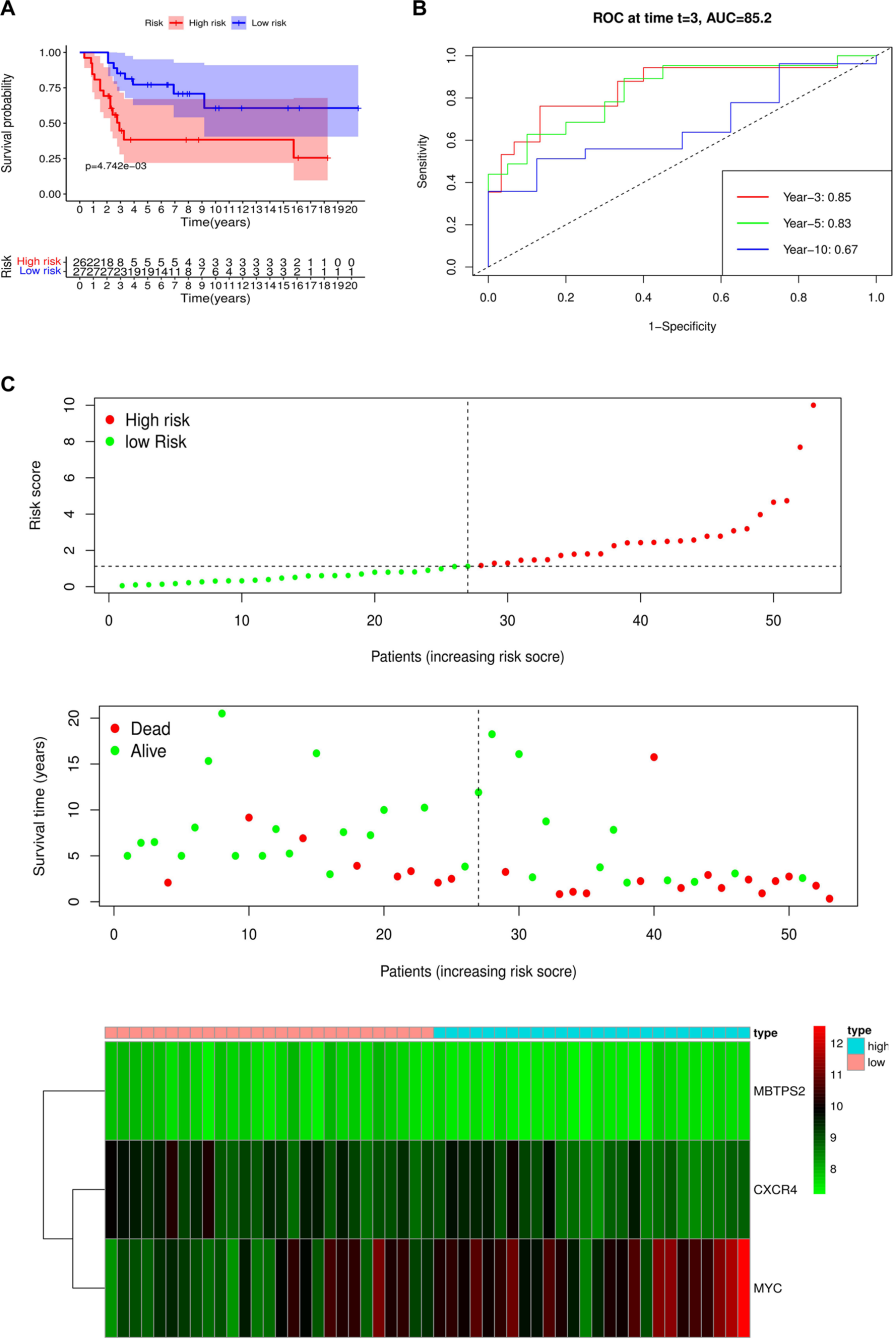
Risk score analysis of the prognostic signature in the training cohort. **A**: Kaplan–Meier survival for low-risk group and high-risk group. **B**: Time-dependent ROC curve at 3, 5, and 10 years for predicting OS based on risk score. **C**: Risk score distribution, survival status, and expression heat map

According to the constructed model formula, a risk score was calculated for each patient, and then the patients were divided into high- or low-risk groups depending on the median score. To further evaluate the predictive performance of the prognostic model in osteosarcoma patients, we performed K-M survival analysis and time-related ROC analysis in the training cohort. The results showed that, based on the prognostic model, the OS of osteosarcoma patients in the high-risk group was shorter than low-risk group (P < 0.05) (Fig. 2A). In addition, as shown in Fig. 2B, the AUC values of the ROC curves for 3, 5, and 10-year survival were 0.85, 0.83, and 0.67, respectively, which indicated a favorable predictive accuracy of the prognostic model. The distribution of the risk score, survival status and corresponding heatmap of the expression level of ARGs of osteosarcoma patients in the training cohort was displayed in Fig. 2C.

**Fig. 3:**
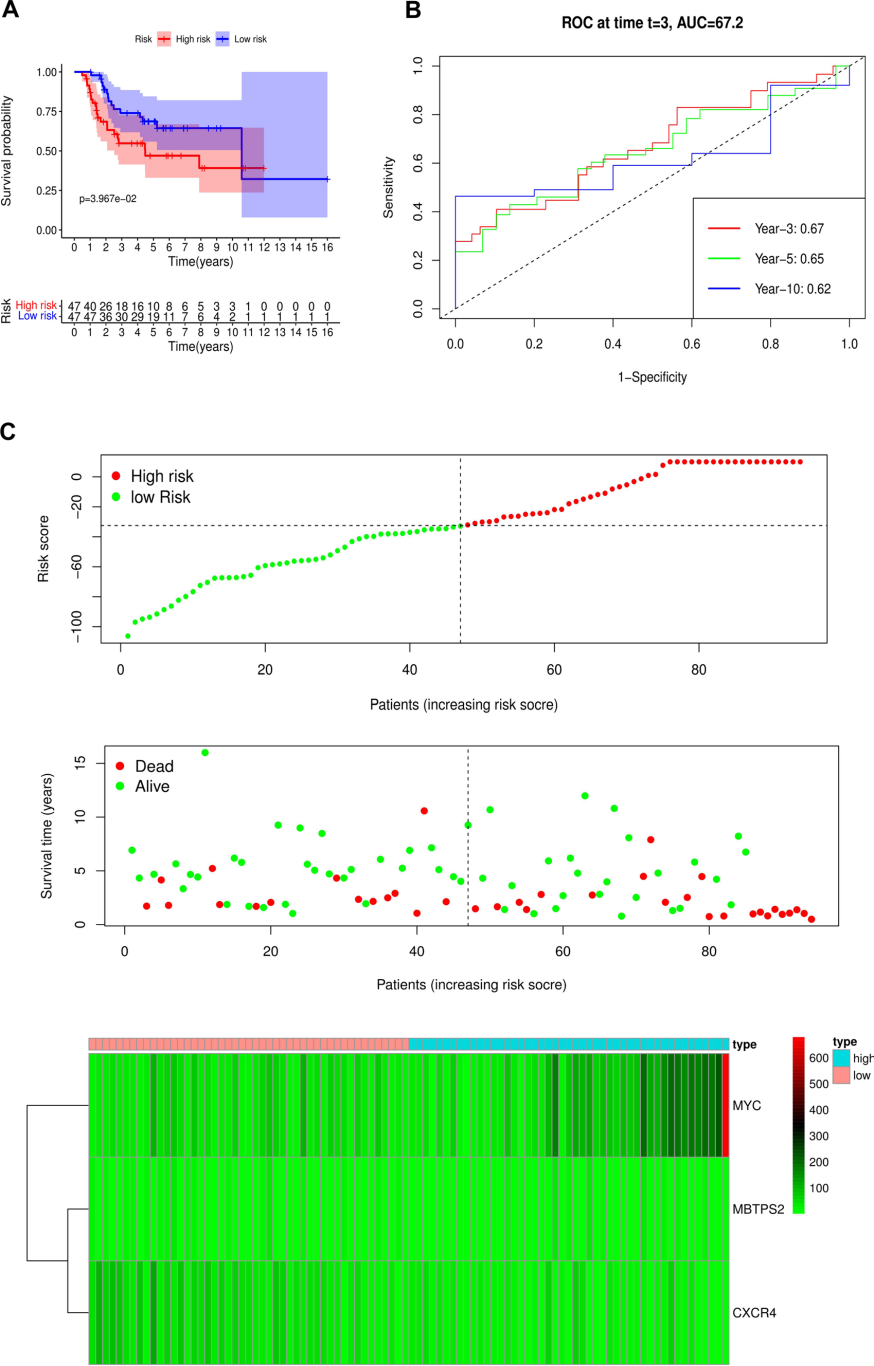
Risk score analysis of the prognostic signature in the verification cohort. **A** Kaplan–Meier survival for low-risk group and high-risk group. **B** Time-dependent ROC curve at 3, 5, and 10 years for predicting OS based on risk score. **C** Risk score distribution, survival status, and expression heat map

To assess the robustness of the model in OS prediction of osteosarcoma patients, we further examined it in the verification cohort from the TARGET database using the same formula. The results revealed that the OS of patients with high-risk scores was also worse than those with low-risk scores in the verification cohort (P < 0.05) and the area under the AUC curve at 3, 5, and 10-year were all over 0.6 (Fig. 3A–C). This result was consistent with the training cohort. Overall, our results suggested that the constructed risk model was an effective prognostic marker for osteosarcoma patients.

### Exploration of the relationship between clinical features and ARGs prognostic signature

**Fig. 4:**
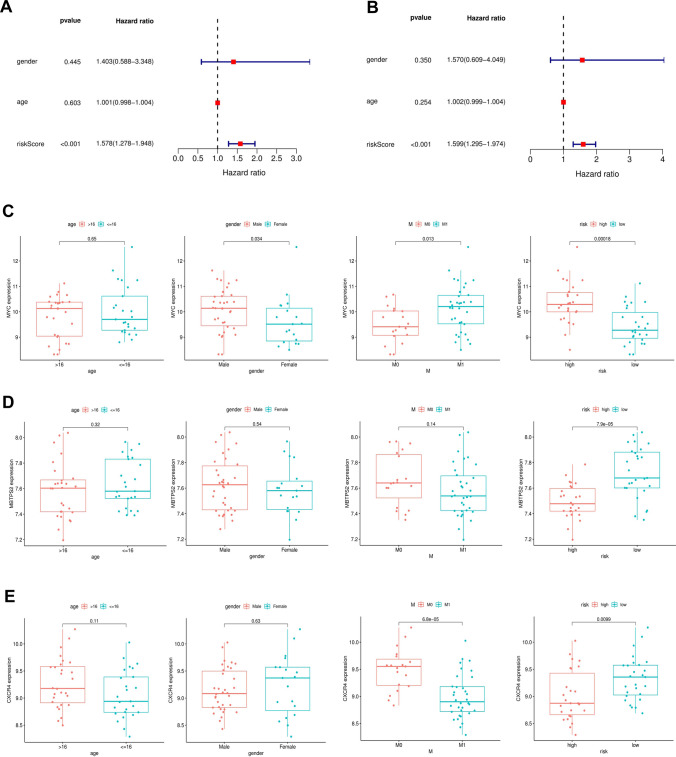
Relationships between three ARGs and clinical parameters in the training cohort. **A**, **B** Univariate and multivariate cox regression analyses revealed the risk score was the independent prognostic factor in the training cohort. **C** Boxplots of the relationship between MYC expression and clinical characteristics. **D** Boxplots of the relationship between MBTPS2 expression and clinical characteristics. **E** Boxplots of the relationship between CXCR4 expression and clinical characteristics

To assess whether the model could serve as an independent prognostic indicator, we carried out univariate and multivariate Cox regression analyses on survival and clinical data from the training cohort. Forest plot results showed that risk score was significantly associated with OS in osteosarcoma patients and was independent of age and gender (Fig. 4A, B). These findings illustrated that the risk score based on three ARGs could be considered an independent prognostic factor for osteosarcoma patients. Afterward, correlations between clinical features and genes within the model were investigated. In the training cohort, we could observe that the MYC expression levels were significantly correlated with gender, metastasis, and risk score of osteosarcoma patients. Both the metastatic group and the high-risk score group showed high expression of MYC. In contrast, the expression levels of MBTPS2 and CXCR4 were lower in both the metastatic group and the high-risk score group (Fig. 4C–E).

Since we were more interested in MYC and MBTPS2, we further verified their expression in osteosarcoma and their correlation with osteosarcoma metastasis in vitro*.*

### Validation of the expression levels of MYC and MBTPS2 in osteosarcoma tissues and cells

**Fig. 5:**
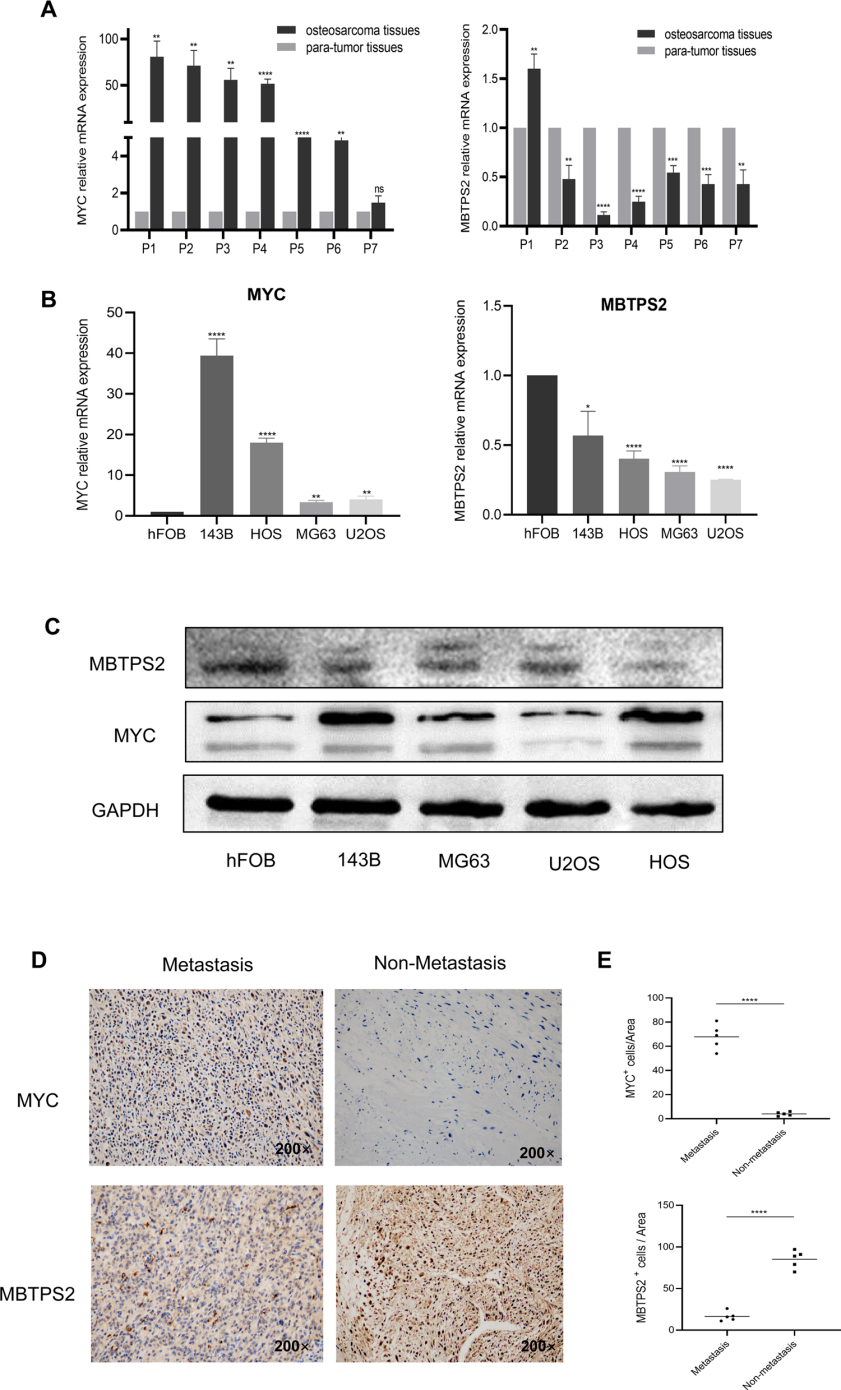
The expression levels of MYC and MBTPS2 in human osteosarcoma samples and cell lines. **A**: The mRNA expression levels of MYC and MBTPS2 in seven osteosarcoma patients. **B**, **C**: The mRNA and protein expression levels of MYC and MBTPS3 in hFOB and four osteosarcoma cell lines (143B, HOS, MG63, U2OS). **D**: Representative pictures of IHC staining for MYC and MBTPS2 in human osteosarcoma samples. **E**: Comparison of MYC- and MBTPS2- positive cells among the 2 groups. Mean ± SD (*P < 0.05, **P < 0.01, ***P < 0.001, ****P < 0.0001)

Initially, we detected the mRNA expression levels of two autophagy-associated genes, MYC and MBTPS2, in osteosarcoma patients’ tissues through RT-qPCR. The results indicated that osteosarcoma samples showed higher MYC mRNA expression levels than that of corresponding adjacent nontumorous tissues, while MBTPS2 expression levels were relatively low in tumor samples (Fig. 5A). In cell experiments, similar results were found that MYC and MBTPS2 were differentially expressed in hFOB and osteosarcoma cell lines at the mRNA and protein levels (Fig. 5B, C). Moreover, the results of IHC demonstrated that MYC expression was up-regulated in the metastatic osteosarcoma patient, and MBTPS2 showed relatively high expression level in the non-metastasis patient (Fig. 5D, E), which suggested the relationship between autophagy and metastasis in osteosarcoma.

### MYC knockdown and MBTPS2 overexpression inhibit osteosarcoma metastasis by inhibiting autophagy in osteosarcoma cells

**Fig. 6:**
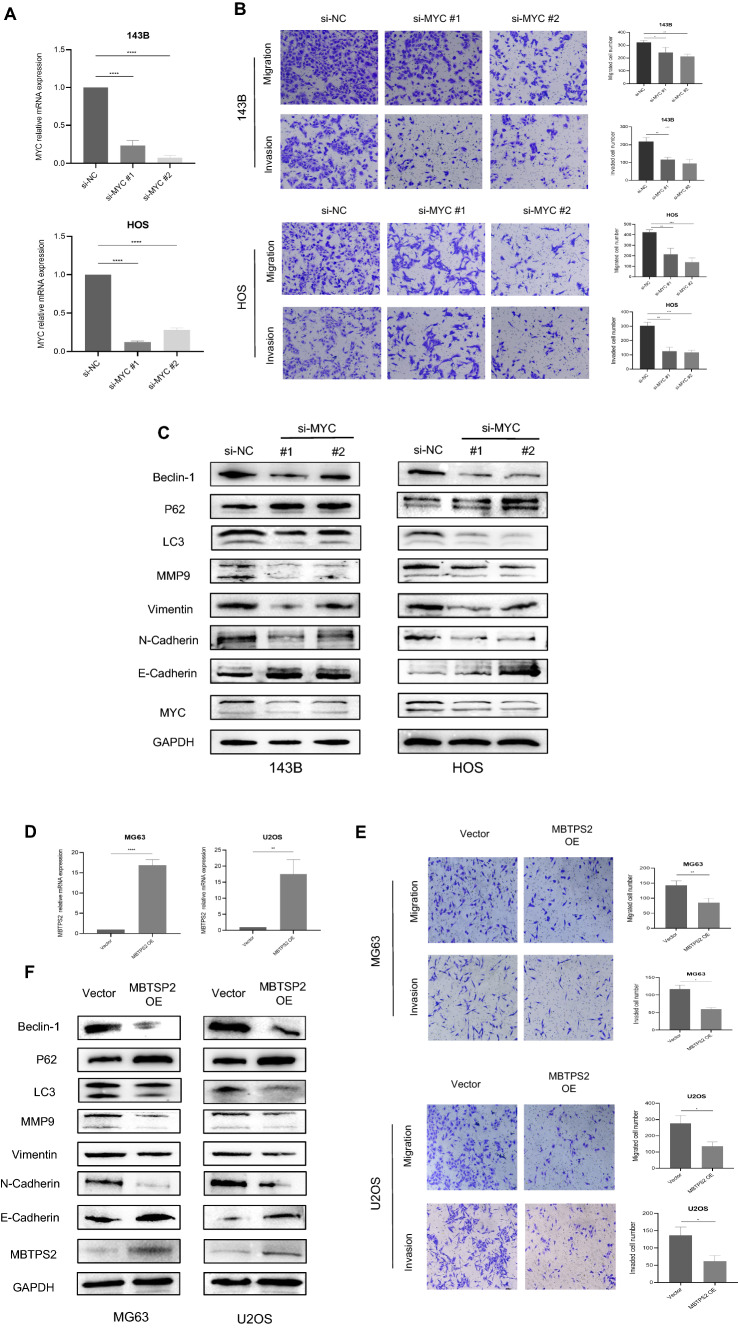
MYC silencing and MBTPS2 overexpression inhibited the migration and invasion of osteosarcoma cells. **A**, **D**: Detection of MYC knockdown (**A**) and MBTPS2 overexpression (**D**) efficiency by RT-qPCR. **B**, **E**: The effects of MYC silencing (B) and MBTPS2 overexpression (**E**) on migration and invasion were evaluated with Transwell assay. Magnification:100 × . **C**, **F**: EMT and autophagy signalling pathways assessment with WB after MYC knockdown (**C**) and MBTPS2 upregulation (**F**). Mean ± SD (*P < 0.05, **P < 0.01, ***P < 0.001, ****P < 0.0001)

To further validate the biological functions of MYC and MBTPS2 in osteosarcoma cells, we depleted MYC expression in 143B and HOS cells with siRNA technology, and elevated the expression levels of MBTPS2 in MG63 and U2OS cells by overexpression plasmid. The knockdown efficiency of MYC gene was verified by RT-qPCR (Fig. 6A). Given the intricate connection between autophagy and metastasis, we next examined the effects of MYC silencing and MBTPS2 overexpression on metastasis ability of osteosarcoma cells by Transwell assay. As shown in Fig. 6B, after MYC silencing, the number of stained 143B and HOS cells decreased significantly. Moreover, the results of immunoblotting demonstrated that the protein levels of N-cadherin, Vimentin, and MMP9 were downregulated, while E-cadherin was upregulated after MYC knockdown. Meanwhile, the two si-MYC groups exhibited decreased LC3 and Beclin-1 protein levels but increased p62 levels (Fig. 6C). Similar results were obtained in osteosarcoma cells with overexpressed MBTPS2 (Fig. 6D–F). MBTPS2 overexpression suppressed the migration and invasion abilities of MG63 and U2OS cells (Fig. 6E). Besides, the autophagy declined after overexpressing MBTPS2 in MG63 and U2OS cells (Fig. 6F). Collectively, these results demonstrated that MYC and MBTPS2 were involved in the metastasis of human osteosarcoma cells by affecting autophagy, which was consistent with our findings based on the public databases.

## Discussion

Osteosarcoma is the most frequent primary malignant bone tumor with an inclination to the metaphysis of children’s and adolescents’ long bones [1, 17]. Due to the tumor heterogeneity derived from genetic instability, 15% to 20% of osteosarcoma patients have clinically detectable distant metastases at the time of presentation, and more than 85% of metastases occur in the lung [18]. At present, the treatment of osteosarcoma is mainly based on surgery and chemotherapy, and the prognosis of the disease is closely related to whether it is combined with lung metastasis. It was reported that the cure rate for osteosarcoma patients who had focal tumors increased up to 70% while the 5-year OS in metastatic cases remained only about 20% [19, 20]. Therefore, it is urgent to develop accurate and reliable prognostic biomarkers to stratify osteosarcoma patients and guide individualized treatment to improve their prognosis [21].

Autophagy is a cytoprotective mechanism which can degrade intracellular damaged organelles and proteins, thereby maintaining cellular homeostasis. However, excessive autophagy can also cause excessive degradation of cytoplasm and organelles, leading to cell death [22]. Increasing evidence in the literature indicated that autophagy played multiple roles in the occurrence, metastasis and chemotherapy resistance of osteosarcoma [15, 23, 24]. Therefore, we constructed an ARGs-based prognostic risk model in our study, which will provide new insights into individualized therapy strategies for osteosarcoma patients.

In the beginning, we downloaded a list of 210 ARGs and extracted their expression levels from the GSE21257 cohort. Univariate Cox regression screened out 11 ARGs associated with prognosis, including ATF4, ATG4A, CD46, CXCR4, GOPC, MAPK1, MBTPS2, MYC, NBR1, PEA15, and SERPINA1. Multivariate Cox regression analysis further identified a risk model consisting of 3 ARGs which could classify osteosarcoma patients into high- and low-risk groups. The KM survival curve indicated that the risk score was negatively correlated with patients’ survival rates and the AUC values of ROC curves revealed that this model was reliable. More importantly, we validated the risk model in the verification cohort and its results confirmed the model's reliability and accuracy. Subsequent univariate and multivariate regression analyses indicated that the risk model was an independent predictor of osteosarcoma patients. We also evaluated the correlation of the individual gene in the model with the clinical characteristics of osteosarcoma patients, and found that the three genes were all associated with osteosarcoma metastasis.

Based on the results above, MYC and MBTPS2 were selected from high- and low- expression gene groups for further exploration. MYC is a multifunctional oncogene that is highly expressed in various human tumors and its mutations were detectable in more than 10% of osteosarcoma patients [25, 26]. In this study, we found that MYC was highly expressed in osteosarcoma tissues and cell lines. More important, IHC results showed MYC expression was up-regulated in metastatic osteosarcoma patients. After MYC knockdown, the metastasis ability of 143B and HOS cells were inhibited, which was consistent with previous studies [27, 28].

MBTPS2, on the X-chromosome, encodes site-2 protease (S2P), is a zinc metalloprotease involved in the regulation of intramembrane proteolysis. MBTPS2 missense mutations were previously identified in dermatological diseases including Ichthyosis Follicularis, Atrichia, Photophobia (IFAP), and Keratosis Follicularis Spinulosa Decalvans (KFSD) [29–31]. Lindert et al. subsequently found that MBTPS2 deficiency could cause an X-linked recessive osteogenesis imperfecta [32]. However, the role of MBTPS2 in osteosarcoma remains unknown. Our experimental results showed confirmed low-expression levels of MBTPS2 in osteosarcoma and its overexpression attenuated the migration and invasion of MG63 and U2OS cells. It is the first study to point out the association between MBTPS2 and osteosarcoma.

It is well known that Beclin-1, P62 and LC3 are autophagy-related proteins [33]. When MYC was knocked down or MBTPS2 was overexpressed, the expression of Beclin-1 and LC3 was suppressed, while the P62 expression increased. These findings suggested that autophagy in osteosarcoma cells was inhibited, which was consistent with previous studies [34–36]. Conclusively, we confirmed the correlation of MYC or MBTPS2 with autophagy and metastasis in osteosarcoma, which provides further verification of the validity of the model. However, some limitations existed in our study. Firstly, gene expression data and clinical profile information was downloaded from open-access databases but not from our clinical data. Besides, the sample sizes of the training and verification groups were relatively small. Moreover, our research was a retrospective study and the conclusion warrants further validation in follow-up researches.

## Conclusion

In conclusion, we established a prognostic model based on three ARGs in osteosarcoma, and it was valuable in accurately predicting osteosarcoma prognosis. More importantly, we verified that autophagy-related genes, MYC and MBTPS2, were closely related to tumor cells metastasis in osteosarcoma. Therefore, they can be viewed as an attractive target with promising therapeutic potential for osteosarcoma.

## Supplementary Information

**Additional file 1**: **Table S1.** Clinical Characteristics of seven osteosarcoma patients.

**Additional file 2**: **Table S2.** The Primer Sequences of Genes.

## Data Availability

All the data involved in this study are available from the corresponding author upon reasonable request.

## Abbreviations

ARGs: Autophagy-Related genes
OS: Overall survival
HADb: Human autophagy database
RNA-seq: RNA-sequencing
TARGET: Therapeutic applications research to generate effective treatments
RT-qPCR: Quantitative Real-Time PCR
IHC: Immuno histo chemistry
HADb: Human autophagy database
AIC: Akaike information criterion
ROC: Receiver operating characteristic
Hfob: Human osteoblast cell line
RT: Room temperature
MBTPS2: Membrane-bound transcription factor protease, site-2
S2P: Site-2 protease
IFAP: Ichthyosis follicularis, atrichia, and photophobia
KFSD: Keratosis follicularis spinulosa decalvans
EMT: Epithelial-Mesenchymal transition
