# Supplementary material for: Construction and validation of a prognostic model for osteosarcoma patients based on autophagy-related genes
Source: Discov Oncol. 2022 Dec 31;13:146. doi: 10.1007/s12672-022-00608-9 (PMC9805482; doi:10.1007/s12672-022-00608-9)
Supplement: Supplementary file 1 — Additional file 1: Table S1. Clinical Characteristics of seven osteosarcoma patients. [file 12672_2022_608_MOESM1_ESM.docx]

| **Clinical Characteristics of OS patients** | | | | | |
| --- | --- | --- | --- | --- | --- |
| No | Age | Gender | Location | Pathology | Metastasis |
| 1 | 41 | Male | Left tibia | Osteosarcoma | No |
| 2 | 17 | Male | Right tibia | Osteosarcoma | No |
| 3 | 8 | Male | Right femur | Osteosarcoma | No |
| 4 | 12 | Female | Right femur | Osteosarcoma | No |
| 5 | 30 | Male | Right femur | Osteosarcoma | Back, Right buttock |
| 6 | 12 | Female | Left femur | Osteosarcoma | No |
| 7 | 52 | Male | Left femur | Osteosarcoma | No |
