# Supplementary material for: Construction and validation of a prognostic model for osteosarcoma patients based on autophagy-related genes
Source: Discov Oncol. 2022 Dec 31;13:146. doi: 10.1007/s12672-022-00608-9 (PMC9805482; doi:10.1007/s12672-022-00608-9)
Supplement: Supplementary file 2 — Additional file 2: Table S2. The Primer Sequences of Genes. [file 12672_2022_608_MOESM2_ESM.docx]

| **Pimier Sequences of Genes** | | |
| --- | --- | --- |
| **Gene** | **Forward primier（5’-3’）** | **Reverse primier（3’-5’）** |
| GAPDH | GGAGTCCACTGGCGTCTTCA | GTCATGAGTCCTTCCACGATACC |
| MYC | GGGTAGTGGAAAACCAGCCTC | AGAGGGTAGGGGAAGACCAC |
| MBTPS2 | ATCTGCCAGTGGTTGTGGAG | GCAGCCAAAAGTACACTGCC |

| **Gene** | **Forward primier（5’-3’）** | **Reverse primier（3’-5’）** |
| --- | --- | --- |
| si-MYC #1 | CGUCCAAGCAGAGGAGCAA | UUGCUCCUCUGCUUGGACG |
| si-MYC #2 | AACGUUAGCUUCACCAACAUU | UGUUGGUGAAGCUAACGUUUU |
